# The Effect of Metabolic Surgery on the Complications of Diabetes: What Are the Unanswered Questions?

**DOI:** 10.3389/fendo.2020.00304

**Published:** 2020-05-29

**Authors:** Karl J. Neff, Carel W. Le Roux

**Affiliations:** ^1^Diabetes Complications Research Centre, Conway Institute, University College Dublin, Dublin, Ireland; ^2^Metabolic Medicine, Imperial College London, London, United Kingdom

**Keywords:** metabolic surgery, diabetes, kidney disease, nephropathy, retinopathy, neuropathy

## Abstract

It is now established that metabolic surgery (also known as bariatric surgery or obesity surgery) is an effective treatment for type 2 diabetes. Data from several randomized controlled trials have shown that surgery, when used as an adjunct to best medical therapy, is superior to medical therapy alone in achieving glycaemic and metabolic treatment targets in diabetes care. This has resulting in metabolic surgery being recommended as a treatment option for obesity-associated type 2 diabetes in national and international diabetes care guidelines. While the superior glycaemic effect of surgery is clear, the effect of surgery on the complications of diabetes is not fully understood. There are observational and epidemiological data that indicate a preventative effect in cohorts who do not have complications at baseline, as well as a positive effect on those with established diabetic kidney disease. However, there is a dearth of randomized controlled studies that specifically examine the effect of surgery on the complications of diabetes. Therefore, we should remain cautious in some cases, especially in those with retinopathy or neuropathy, as there is potential for deterioration of disease post-operatively. Further study is needed on this important topic. A lot is known, but there remain several unanswered questions. This article summarizes what we know about the effect of metabolic surgery on the complications of diabetes, poses some unanswered questions, and suggests how we could answer them.

## Introduction

Metabolic surgery (also referred to as bariatric surgery or obesity surgery) is now recommended as a mainstream treatment for type 2 diabetes in people who have both obesity and type 2 diabetes (1). This recommendation, supported by multiple international diabetes organizations including the American Diabetes Association and the International Diabetes Federation, is based on data from randomized controlled trials comparing surgical intervention to best medical therapy (2). In these trials, metabolic surgery has been proven to improve glycaemic control, in the short- and medium-term, to a greater extent than medical therapy alone (2). Surgery is particularly recommended for people with both obesity and type 2 diabetes in whom dietary and medical therapy has been unsuccessful in achieving diabetes treatment targets.

In many cases, the effect of surgery on type 2 diabetes can be so great as to induce remission of disease and normalization of glycaemia. It should be noted that this effect is often transient (3, 4). Therefore, surgery should not be offered as a "cure”' for diabetes, but rather as an effective treatment for type 2 diabetes. Approximately 50% of those who have metabolic surgery and achieve remission of their diabetes will have recurrence of diabetes within the subsequent 10 years (3). However, despite the recurrence of diabetes, these patients usually achieve much better glycaemic and metabolic control than they did before surgery, and so the beneficial glycaemic and metabolic effect of surgery, as compared to medical therapy alone, is retained (3).

However, it should be noted that surgical treatment of diabetes and obesity has associated complications (1, 2). The specific surgical complications are procedure dependent, but all surgical recipients will be at risk of hospitalization for treatment of complications of surgery, and will remain at high risk of micronutrient malnutrition, with the potential for associated anemia or neuropathy, for life (1). Surgery is not for everyone. An expert multidisciplinary assessment is needed for all surgical candidates to ensure that they are suitable and ready for surgery. Given the highly specialist nature of this work, surgery should only be performed in high volume centers by specialist surgeons who work within expert multidisciplinary teams (1).

The improved glycaemic control achieved with surgery could be expected to reduce the burden of microvascular complications in people with diabetes. However, there are not many randomized controlled trials specifically examining the effect of metabolic surgery on microvascular outcomes. There are experimental models, observational data, and one randomized controlled trial, that support the hypothesis that surgery could have a role in managing not only diabetes, but also its complications. However, many unanswered questions remain.

## Diabetic Kidney Disease

### Diabetic Kidney Disease: What We Know

Type 2 diabetes and obesity often co-exist, and both can contribute to the development of renal dysfunction and disease (5–7). Obesity is associated with proteinuric renal disease even in the absence of diabetes (8). In cohorts with both diseases, there appears to be an additive effect. Having both obesity and diabetes increases the risk of developing renal disease, and a diagnosis of obesity is a key risk factor in the progression of diabetic kidney disease (DKD) (7, 9). Therefore, populations with both obesity and type 2 diabetes should be considered at elevated risk for the development of DKD. Given this pathological synergy, it seems sensible to suggest that in cohorts with both obesity and type 2 diabetes, treatment approaches should be simultaneously targeted at both diseases in order to prevent or treat any associated DKD.

In diabetic kidney disease (DKD), the first clinical manifestation is albuminuria. Metabolic surgery reduces the risk of developing albuminuria in the first instance (10–14). Surgery also results in reduced proteinuria, including albuminuria, even in cases of established disease (15, 16). This effect of surgery is seen within the first 12 months post-operatively, and lasts for at least five years (16–19). This effect is reported in cohorts with and without type 2 diabetes, and can lead to remission of established microalbuminuria in both cohorts (16, 18). A single randomized controlled trial has shown that Roux-en-Y gastric bypass is superior to best medical treatment in causing remission of albuminuria over 2 years, and thus inducing remission of early diabetic kidney disease (JAMA Surg; in press).

While the data for the effect of surgery on both preventing and remediating proteinuria are generally based on measured protein excretion, including 24 h urine collections, almost all of the data on the effect of metabolic surgery on renal function are based on estimated glomerular filtration rate (eGFR) rather than measured glomerular filtration rate (mGFR). It should be noted that in the context of significant weight loss, serum creatinine concentrations usually decrease, resulting in an apparent increase in eGFR (17, 20). Calculators for eGFR have not been validated for use in the context of significant and rapid weight loss, as occurs after metabolic surgery. Therefore, eGFR in this population should be interpreted with caution.

In general, there is no deleterious effect of surgery on eGFR in those with preserved renal function at the time of surgery (19). This seems to be the case for populations both with and without type 2 diabetes at baseline. There are data to suggest a potent renoprotective effect of surgery in people with type 2 diabetes and early DKD. In case-control data comparing metabolic surgery and medical therapy in people with type 2 diabetes, and albuminuria with preserved renal function, surgery was associated with stabilization of eGFR and remission of albuminuria over a 10 year follow-up period, as compared with a steady decline in renal function in the medical group (21).

While there are very few data on the effect of metabolic surgery on people with an eGFR of <60 ml/min/1.73 m^2^ (stage 3–5 chronic kidney disease), the available data indicate potential benefit (17, 19, 22). When using eGFR, there seems to be a stablisation in renal function in recipients of metabolic surgery that can last up to 9 years post-operatively (19, 22–24). Notwithstanding the limitations of eGFR in this cohort, the renal data are reassuring. The maximal weight loss typically occurs within 2 years of surgery. After this point, weight loss stabilizes, and the eGFR calculators are presumably more robust (although this is not proven to be the case).

In clinical practice, metabolic surgery is offered to selected patients on dialysis who require transplantation, but who are excluded from transplant lists solely on the basis of their weight. While this cohort is understandably at high risk of peri-operative and post-operative morbidity and mortality, the benefits of surgery can exceed in the risks in some cases. For selected cohorts, surgery can be risk-acceptable and effective, resulting in successful renal transplantation (25). In some series of patients with end-stage renal disease who have surgery, cases of recovery of renal function in dialysis-dependent patients have been reported (26, 27). In these cases, histological markers of renal disease improved after surgery in parallel with the improvements in renal function.

The mechanisms for the above-described improvement in renal function and prevention of renal disease are not fully understood. Metabolic surgery reduces weight, improves hypertension and ameliorates hyperglycaemia, which can explain some of the effect on eGFR (3, 14, 19, 22, 28, 29). However, other mechanisms, such as resolution of pro-inflammatory pathways, may also contribute to the improvement in markers of renal disease (30, 31). The interplay between obesity and diabetes is difficult to tease out in human studies, but in animal models of DKD the inflammatory effect of obesity seems to be only partially responsible for improvements in proteinuria (30). Therefore, it seems that several mechanisms work together to produce the renal benefits seen in recipients of metabolic surgery.

Glomerular hyperfiltration is a phenomenon seen in obesity related renal disease, with or without co-morbid type 2 diabetes. Hyperfiltration results in glomerular hypertrophy, and the increased rate of filtration is associated with oxidative stress and activation of pro-inflammatory pathways. In some cases, the end-point of chronic hyperftltration is focal segmental glomerulosclerosis, with an associated clinical presentation of proteinuric renal disease termed obesity related glomerulopathy, which is similar in clinical presentation to diabetic kidney disease, but which can occur in the absence of diabetes or hyperglycaemia (8). This condition can result in end stage renal failure.

Obesity related glomerulopathy is a result of a confluence of pro-inflammatory pathways, ectopic lipid accumulation within the kidney, and podocyte dysfunction in the context of insulin resistance and oxidative stress (8). As it can occur in the absence of diabetes and hypertension, it is a clear demonstration of the importance of multiple non-glucocentric mechanisms that are involved in the onset and progression of diabetic kidney disease in those with type 2 diabetes and obesity (8). As is the case in diabetic kidney disease, metabolic and bariatric surgery can ameliorate obesity related glomerulopathy by remediating inflammation and reducing hyperfiltration (8).

### Unanswered Questions in Diabetic Kidney Disease

#### GFR Accuracy—Do the Estimates Measure Up?

An essential question to answer is whether the estimated GFR calculators can be relied upon after metabolic surgery. Measured GFR before and after surgery appears stable when corrected for body surface area (17, 20, 32–36). However, in these studies there were very few participants with diabetes, and follow-up was less then 24 months in almost all cases.

This is problematic as we are basing our current ideas around the use of metabolic surgery in DKD on the effect of eGFR [usually estimated with the Modification of Diet in Renal Disease (MDRD) formula] in long-term epidemiological and cohort studies. The MDRD formula has been shown to be inaccurate in cohorts with type 2 diabetes and obesity (37, 38). Part of the problem is the presumption of a body surface area of 1.73 m^2^, which is an underestimate in many people with obesity (38). However, even in longitudinal studies of measured GFR, concerns around eGFR calculators exist with respect to their validity in people with type 2 diabetes (39).

This issue is compounded by the changes in muscle mass that occurs after metabolic surgery (40). Serum creatinine is primarily derived from muscle mass, and so calculators based on serum creatinine may be prone to error if we compare eGFR before and after significant weight loss. The loss of muscle mass only appears to be significant in the first year after surgery, with apparent stabilization of muscle mass from the end of the first post-operative year (40). While the studies of measured GFR completed in the first year post operatively have been reassuring, very few participants were followed beyond the weight loss phase (17, 20–22). Therefore, it is unknown if the current creatinine based calculators for eGFR are valid in people with type 2 diabetes following metabolic surgery.

Calculators for eGFR other than MDRD can be used, although none appear to be as accurate as we would like (33). This is especially true in the weight loss phase in the first year after bariatric or metabolic surgery (33, 34, 37). Of the currently available calculators, CKD-EPI is the most accurate (33). CKD-EPI can underestimate GFR in people with obesity before surgery, and overestimate it in the post-operative phase, but the use of Cystatin C as a marker of filtration can enhance the accuracy of CKD-EPI (33, 34). However, Cystatin C is not widely available for use, and remains outside of standard clinical practice at present. While the estimated values may not be entirely accurate in any of the calculators, the trends over time do parallel, and so whatever calculator is used at the onset, should be consistently used over time to detect any significant changes in eGFR (22).

Given the above, in our opinion, it seems that that new studies are needed. These should be validation studies of eGFR calculators in populations with type 2 diabetes and obesity. Two cohorts are needed in these studies; a post-operative population who had metabolic surgery over 2 years before entry into the study, and a cohort matched for age, weight, and relevant comorbidities and medication use. It should be noted that given the dynamic physiological changes associated with surgery, eGFR calculation may not be able to achieve acceptably accurate results. Direct measurement of GFR is the only way to really gauge the effect of metabolic and bariatric surgery on renal function and disease. While the complexity of the measurement process means that measured GFR remains inaccessible for standard clinical care, the use of simplified direct measurement of GFR may prove to be the future of renal surveillance in this cohort.

#### Is Metabolic Surgery Better Than Medical Management for Treating People With DKD and an eGFR of <60 ml/min/1.73 m^2^?

There is abundant evidence that metabolic surgery is more effective than medical therapy alone in treating type 2 diabetes. There is also evidence that it can treat hypertension more effectively than with medication alone, although this evidence base is not as well-developed (3, 41). Given that these two major risk factors are well-treated by metabolic surgery, it seems logical to presume that surgery would be more effective than medical therapy in treating established DKD.

However, it remains the case that we still do not have any randomized controlled data from trials specifically powered to detect treatment effect differences between surgery and medical therapy in DKD with an eGFR <60 ml/min/1.73 kg/m^2^. This is particularly important in the longer-term in people with renal impairment at baseline. While glycaemic control and blood pressure improve shortly after surgery, and remain improved in the medium to long-term, there is an increased risk of nephrolithiasis in surgical recipients (42). Oxalate nephropathy may develop after metabolic surgery, predisposing to nephrolithiasis and chronic renal disease (43). This risk appears to be procedure dependent, but is not well-quantified. However, it may be that in people with eGFRs of <60 ml/min/1.73 kg/m^2^ the risk/benefit balance is not that strongly in favor of surgical treatment.

On balance, it seems likely that the potential renal benefits would exceed the risks. However, this needs to be specifically studied. Randomized controlled trials of surgery and best medical therapy are needed in people with established DKD and eGFR of <60 ml/min/1.73 m^2^. These studies should incorporate a treat-to-target protocol, so that the medical arms achieve equivalent glycaemic and blood pressure targets. Best medical therapy should include renin-angiotensin-aldosterone system inhibitor or blockade, and sodium glucose co-transporter 2 inhibitor therapy. They should also include longer-term follow-up and measurement of urinary markers of nephrolithiasis.

## Diabetic Retinopathy

### Diabetic Retinopathy: What We Know

There is little evidence to inform us on the effect of metabolic surgery on diabetic retinopathy. From the existing data, it seems that most people who do not have retinopathy pre-operatively, do not go on to develop it post-operatively (44–46). For those with pre-existing retinopathy, most either remain stable, or improve, in disease severity (44–46). However, some do deteriorate and develop more severe, and potentially sight-threatening, disease (44, 45, 47).

The existing longitudinal data on microvascular outcomes suggest that in most people surgery prevents the development or progression of diabetic retinopathy (11, 13, 14, 29). While none of these studies were specifically powered to examine the effect of surgery on retinopathy, these long-term real-world data are reassuring as they show that surgery is associated with significant reductions in the risk of developing retinopathy without a strong signal for significant deterioration of disease at population level (11, 13, 29).

This makes physiological sense, as surgery is proven to be more effective that medical therapy alone in helping people with type 2 diabetes meet glycaemic treatment targets (28, 48, 49). Hyperglycaemia is the major risk factor for the development and progression of retinopathy (50). Therefore, ameliorating hyperglycaemia with surgery would be expected to be protective against retinopathy.

However, while the long-term effect may be expected to be protective, the immediate post-operative period should be considered to be a time when the individual is at elevated risk of disease progression. This is due to the rapid improvement in glycaemic control seen after surgery. Rapid improvement in hyperglycaemia has long been associated with progression of retinopathy (51). Metabolic surgery results in an improvement in hyperglycaemia in the immediate post-operative period, with significant changes in HbA1c within months of treatment (48). This rapid improvement should be expected to put the surgical recipient at risk of progression of disease.

The existing evidence base is almost exclusively limited to cohort studies and uncontrolled case series. The populations studied are heterogenous with varying degrees of pre-operative disease and post-operative follow-up. There are very few randomized controlled data available on retinopathy. In the STAMPEDE trial, where 150 participants were randomized to sleeve gastrectomy, Roux-en-Y gastric bypass, or best medical therapy, formal scoring of retinopathy was not published, but it was reported that there was no significant change in retinopathy disease severity in any of the treatment groups (28).

However, the concern about the immediate post-operative effect of surgery on retinopathy is borne out in some of the published case series documenting the effect of surgery on retinopathy (44, 45, 47). In these series, many cases of disease progression have been described. Given the design of these studies, we cannot be clear as to the major risk factors for progression of disease, but it does seem that pre-operative hyperglycaemia is likely to be a major risk factor. In one study, the authors noted a difference in pre-operative HbA1c of over 11 mmol/mol (1%) between those who had progression of disease post-operatively and those who did not (47). The mean change in HbA1c was comparable between groups, but pre-operative HbA1c concentrations were higher in those who had post-operative progression of retinopathy (47).

In some cases, progression of disease can be sight threatening. From the reported cases, it appears to be a combination of pre-operative HbA1c, magnitude of change in HbA1c concentration post-operatively, and presence of pre-existing retinal disease, that is most important in determining the risk of progression to sight-threatening disease following surgery (44, 47, 52). Therefore, while in general surgery could be expected to prevent the development or progression of retinopathy, caution must be taken in candidates for surgery who have pre-existing retinopathy, especially if they are significantly off glycaemic targets pre-operatively.

### Unanswered Questions in Retinopathy

#### What Is the Effect of Metabolic Surgery on Retinopathy?

The case series and cohort data outlined above suggest a rate of post-operative deterioration in retinopathy of between 1 and 45%. (46, 47, 52–56). This wide range is likely explained by the heterogeneity of the cohorts, both in terms of clinical characteristics and size. Different grading systems were also used across studies, which critically limits comparison between study cohorts.

This illustrates the need for robust prospective large-scale cohort studies examining the effect of surgery on retinopathy specifically. Any such study should include people with type 2 diabetes, both those with and without retinopathy, who are followed up after surgery with a robust validated grading system to determine if there is any change before and after surgery. In order to control for background rates of progression of retinopathy, the surgical cohort should be matched with a control cohort. Adequate matching will require a cohort with similar glycaemic control at baseline, comparable retinal grading at baseline, and equivalent duration of diabetes.

Follow-up for any study of retinopathy needs to be up to 5 years at least, as there can be changes in retinal scores for up to 4 years post-operatively (47). Therefore, even in those who achieve treatment targets, or even remission of diabetes, after surgery, a risk of progression of disease may exist. In the absence of definitive evidence, caution is best, and so in current practice, retinal screening should continue in those who have had surgery for at least 4 years. A longer-term study would inform us to the need for such an approach.

#### Are There Risk Factors for Development or Progression of Retinopathy After Metabolic Surgery?

There are signals in the existing cohort studies that pre-operative HbA1c concentrations, and grading of pre-existing retinopathy, are both important risk factors for progression of disease post-operatively. However, this needs to be proven and defined. What are the risk factors that are important in predicting progression? And what are the thresholds that define high-risk from normal risk? A well-designed prospective case control study could answer these questions.

In most studies, higher pre-operative HbA1c concentrations are associated with a higher rate of post-operative progression of retinopathy (47, 52, 54). The HbA1c threshold at which retinal risk really accelerates is not known, but in the cohort study with the highest rate of deterioration in retinopathy after surgery (9 people out of 20), the mean pre-operative HbA1c was 81 mmol/mol (9.5%) (54). In one study, the authors defined an increased risk of higher grades of post-operative retinopathy with every 1 mmol/mol increase in pre-operative HbA1c (47). This needs to be verified in a larger study.

As well as baseline HbA1c, the rapid change in HbA1c concentrations that often occur after surgery may be associated with progression of retinopathy. In this case, the cohort data have shown conflicting results. In some cohorts, there is a clear trend for deterioration in those who achieve the greatest improvement in HbA1c (52). In others, there is no such trend (47). However, the differing design of the studies, and differences in baseline characteristics between cohorts, explain this conflict. Based on the evidence for retinal disease progression with rapid improvements in glycemic control in other settings, it seems very likely that rapid changes in glycaemic control will prove to be a risk factor for progression of retinopathy. However, this remains to be proven. Theoretically, this risk factor could be modified with anti-diabetes medical therapy prior to surgery, in an effort to reduce HbA1c pre-operatively, thus reducing the rapid slope of change immediately after surgery.

There is no clear threshold for risk of post-operative progression of retinopathy. While baseline hyperglycaemia is clearly important, rapid improvement of hyperglycaemia is also important (51). Given what we know so far a pre-operative HbA1c of over 75 mmol/mol (9%) and a change in HbA1c of over 11 mmol/mol (1%) before and after surgery, seems reasonable as markers of risk (47, 52, 54). However, further study is needed to clearly define these glycaemic risk thresholds.

Recent data have suggested there are many phenotypes of type 2 diabetes that could be mapped genetically, and potentially via proteomic or metabolomics profiles (57). It is known that likelihood of remission of type 2 diabetes can be partially predicted by pre-operative factors including HbA1c and C-peptide concentrations (58). Using these parameters with other biochemical, metabolic, and anthropometric factors in multivariate models may be able to predict those who will have a rapid glycaemic response to surgery (59).

The other likely risk marker of post-operative deterioration in retinopathy is pre-operative grade of disease. It seems that any grade of retinopathy, no matter how early, increases the likelihood of higher grades post-operatively (47, 52). Most people who do not have retinopathy pre-operatively remain disease free after surgery (44–46). In those who do have deterioration of disease, there is no clear relationship between grade for pre-operative disease and eventual post-operative grading. Cases of severe and potentially sight-threatening disease have been described in people with minimal retinal disease pre-operatively (44, 45, 47). These cases are confounded by other changes in characteristics, predominantly glycaemic change, over time, and so the importance of the grade of retinal disease itself as a risk factor remains unclear.

Therefore, a large long-term case control study is needed, where people who go on to have metabolic surgery are well-characterized at baseline and then followed over at least 5 years. The surgical cohort should be matched to a control cohort for all relevant risk factors. Given the likely confounding effect of improvement in glycaemic control, statistical input should be sought as how to delineate the glycaemic effects from the effect of pre-existing grade of retinal disease and other potential risk factors.

While awaiting the results of any future study on retinopathy, it is best to be cautious. Therefore, we recommend pre-operative retinal assessment for all patients with type 2 diabetes who go on to have bariatric or metabolic surgery. Reassessment should be scheduled post-operatively. If there is any retinopathy, then reassessment should occur within three to 6 months post-operatively. If pre-proliferative retinopathy, or any maculopathy, is detected, then specialist ophthalmology advice should be sought to determine if pre-operative treatment is needed.

## Diabetic Neuropathy

### Diabetic Neuropathy: What We Know

There are even fewer data on the effect of metabolic surgery on diabetic neuropathy than there are on the effect of surgery on retinopathy. However, the data that do exist demonstrate that metabolic surgery is associated with a reduced risk of developing diabetic neuropathy (13, 14, 28, 29, 60). These data were all derived from subjective reporting of neuropathy, either by patients themselves or from healthcare providers. In a small study using nerve conduction studies, there was no change in electrophysiological markers of neuropathy before and after surgery (46).

It is reasonable to expect that neuropathy would be less likely to occur after surgery given the significant improvement in glycaemic control induced by metabolic surgery. Therefore, the available data agree with expectation. However, there are very few people included in these studies with pre-existing neuropathy or diabetic foot disease. There is some cause for concern that surgery may result in treatment-induced neuropathy given the rapid rate of improvement in glycaemic control (61, 62).

### Unanswered Questions in Neuropathy and Diabetic Foot Disease

Metabolic surgery can result in neuropathy, even in the absence of diabetes (63). The etiology of neuropathy after surgery varies, but can be related to deficiencies in micronutrients such as vitamin B12. While there is no evidence that treatment induced neuropathy is a common phenomenon after metabolic surgery, cases of serious diabetic neuropathy and arthropathy have been described in the context of rapid improvements in glycaemic control and weight (61, 62). Therefore, it will be necessary to study the effect of metabolic surgery on neuropathy, especially in people with pre-operative disease, in order to quantify the risk of post-operative deterioration.

As well as quantifying the effect of surgery on neuropathy, prospective cohort studies are needed to delineate the important mechanisms that could be associated with post-operative neuropathy. There are multiple potential insults associated with surgery. The rapid improvement in glycaemic control may induce a treatment-induced neuritis. Micronutrient deficiencies can also produce neuropathy. Anecdotal evidence would suggest that those that present with late onset of neuropathy, often many years after surgery, are those who are not taking regular comprehensive micronutrient supplementation. Specifically, deficiencies in B vitamins may be critical factors in the development of neuropathy. Therefore, any such study must be designed to comprehensively gather all the relevant data to allow a full analysis of all potential risk factors.

Diabetic foot disease is a source of major morbidity in people with type 2 diabetes. Neuropathy is a major contributor to diabetic foot disease. Therefore, if there was a risk of deterioration in neuropathy post-operatively, this may worsen existing diabetic foot disease or increase the risk of developing diabetic foot disease. There are no studies on this topic, but given the risk of harm in people with active diabetic foot disease (ulceration or arthropathy), there is an urgent need to investigate the effect of surgery on nerve function, both in those without neuropathy at baseline, and those with active diabetic foot disease.

## Cardiovascular Disease

### Cardiovascular Disease: What We Know

Metabolic surgery significantly improves blood pressure and cholesterol concentrations in tandem with improved glycaemic control in type 2 diabetes. Therefore, is it not surprising to note that surgery is consistently associated with reduced rates of myocardial infarction and stroke (4, 14, 64). This reduces the cardiovascular mortality rate in these cohorts by ~50%.

While the observational and epidemiological evidence are very convincing, it is not conclusively proven that surgery would be more effective than medical therapy in reducing cardiovascular mortality. There are no randomized controlled trials of surgery and best medical care where cardiovascular morbidity or mortality are primary outcomes. These studies remain to be completed, and should be focused on those with increased cardiovascular risk in the first instance.

### Unanswered Questions in Cardiovascular Disease

#### Can We Determine Who Is Likely to Benefit Most From Metabolic Surgery?

It may be that there are certain cohorts who would benefit more from surgery than others. Those with existing cardiovascular disease may not be good surgical candidates. However, if metabolic surgery was being considered in order to reduce cardiovascular risk, it would be useful to know who would be most likely to benefit from intervention.

As mentioned above, there are different clinical phenotypes of type 2 diabetes that can be mapped to genotypes (57). It may be that some of these phenotypes would be more likely than others to respond to surgery in terms of weight loss or improved glycaemic control. However, it may also be the case that there are certain genotypes that would respond better than others in terms of improvements in cardiovascular risk factors. Multivariate models including phenotypic, genotypic, and proteomic parameters may be able to predict those who will have a good response to surgery (59).

Therefore, large-scale population studies are needed. People who go on to have surgery should be characterized genotypically and phenotypically. Samples for proteomic and metabolomics profiles should be taken. Then, as the population ages, and cardiovascular disease becomes apparent, modeling for identifiable risk markers should be completed. This would need to be a longer-term project if completed prospectively, but use of existing bariatric registries could make such a project more feasible (presuming that they included the relevant data).

#### Do Patients With Obesity and Type 2 Diabetes Have a Greater Health Economic Benefit From Surgery With Respect to Cardiovascular Disease?

Metabolic surgery is associated with reduced cardiovascular morbidity and mortality, including reduced rates of hospital admission for cardiovascular disease and heart failure (4, 64, 65). It is clear that in general metabolic surgery is cost-saving not just cost-effective (66). However, this is when people both with and without diabetes are included. A large proportion of the cost-saving is due to prevention of chronic disease, including diabetes and cardiovascular disease, and therefore reduced costs of treatment.

It would be reasonable to expect that there would be a greater cost saving associated with type 2 diabetes given the reduced medication and insulin use that generally occurs post-operatively (28). However, the health economic data are not entirely clear. Some studies indicate a greater cost benefit in people with type 2 diabetes, but others do not find a cost benefit (67, 68). This is likely due to differences in analysis. The lack of cost benefit is due to a greater number of hospital presentations for both routine follow-up care and emergency care (68). However, this is only a cost issue in the first 2 years post-operatively (68). In longer-term follow up, cost savings may be greater.

In cardiovascular disease and diabetes, it may be that there is a major cost saving. In addition to preventing primary events, metabolic surgery can offer secondary prevention and reduce costs associated with coronary artery disease treatment (69). This is not fully defined, and therefore a cost-analysis of people with both diabetes and cardiovascular disease at baseline is needed. It may be that due to a reduced number of secondary events and reduced medication use, people with both type 2 diabetes and cardiovascular disease offer greater health economic benefit to a healthcare system.

## Conclusions

Metabolic surgery is an effective treatment for type 2 diabetes, and given the data available to date, is effective at preventing the development of diabetic complications. However, there is a lack of specific randomized controlled studies on the effect of surgery on the complications of diabetes. These are urgently needed. While waiting for these data, we should exercise care in people with retinopathy, neuropathy and diabetic foot disease, as there is potential for deterioration of disease post-operatively.

As well as physiological and clinical effects, more study is needed on the health economic effects of surgery. It seems logical to assume that people with type 2 diabetes and complications such as diabetic kidney disease or cardiovascular disease would be likely to offer greater cost savings to healthcare systems. Further studies are needed to determine if this is the case.

## Author Contributions

KN and CL both contributed to the literature review and the drafting of this article.

## Conflict of Interest

The authors declare that this paper was completed in the absence of any commercial or financial relationships that could be construed as a potential conflict of interest.
